# Transcriptome analysis of differential gene expression in the longissimus dorsi muscle from Debao and landrace pigs based on RNA-sequencing

**DOI:** 10.1042/BSR20192144

**Published:** 2019-12-04

**Authors:** Shang-Qiao Song, Wei-wei Ma, Su-Xian Zeng, Chao-Long Zhang, Jin Yan, Cui-Cui Sun, Xin Li, Rui-Min Wang, Zong-Qiang Li

**Affiliations:** 1 College of Animal Science and Technology, Guangxi University, Nanning, Guangxi, China; 2 Shajing Subdistrict Office, Jiangnan District, Nanning City Aquatic Animal Husbandry and Veterinary Station, Guangxi; Nanning, Guangxi, China

**Keywords:** Debao pig, GO analysis, KEGG pathway enrichment, qRT-PCR

## Abstract

RNA-seq analysis was used to identify differentially expressed genes (DEGs) at the genetic level in the longissimus dorsi muscle from two pigs to investigate the genetic mechanisms underlying the difference in meat quality between Debao pigs and Landrace pigs. Then, these DEGs underwent functional annotation, Gene Ontology (GO) and Kyoto Encyclopedia of Genes and Genomes (KEGG) pathway enrichment, and protein–protein interaction (PPI) analyses. Finally, the expression levels of specific DEGs were assessed using qRT-PCR. The reference genome showed gene dosage detection of all samples which showed that the total reference genome comprised 22342 coding genes, including 14743 known and 190 unknown genes. For detection of the Debao pig genome, we obtained 14168 genes, including 13994 known and 174 unknown genes. For detection of the Landrace pig genome, we obtained 14404 genes, including 14223 known and 181 unknown genes. GO analysis and KEGG signaling pathway analysis show that DEGs are significantly related to metabolic regulation, amino acid metabolism, muscular tissue, muscle structure development etc. We identified key genes in these processes, such as *FOS, EGR2*, and *IL6*, by PPI network analysis. qRT-PCR confirmed the differential expression of six selected DEGs in both pig breeds. In conclusion, the present study revealed key genes and related signaling pathways that influence the difference in pork quality between these breeds and could provide a theoretical basis for improving pork quality in future genetic thremmatology.

## Introduction

Pigs are an important source of meat production worldwide [1,2]. With improvements in individual living standards, pork plays an increasing number of important roles in citizens’ lives, especially in Chinese food [3]. Therefore, the importance of meat quality cannot be ignored. Accordingly, meat quality has many influencing factors, such as breed and post slaughter handling. Between these influential factors, breed is more important [4–6]. More indigenous pig breeds exist in China than in any other country worldwide [7,8]. In China, 118 indigenous pig breeds are listed on the World Watch List for Domestic Animal Diversity [9]. However, Western commercial pig breeds, over the past several decades, have been artificially selected consistently by breeders and farmers for higher muscle percentage and lower carcass fatness [10], and considerable progress has been made regarding these characteristics. For example, Large White pigs (LW) and Yorkshire pigs have faster growth characteristics and a higher muscle percentage compared with other pigs [11–13].

Chinese indigenous pig breeds, compared with Western commercial pig breeds, have higher IMF, increased tenderness, and better meat quality [14–16]. The Debao black pig (DB), a local black breed with the traits of higher meat quality, lower growth rate, more carcass fatness, and disease resistance [17], is distributed only in the Southeast area of Guangxi Province, China. DB pigs have been fed grains, vegetables, tubers, wild herbs, and so on for centuries. As a traditional fat-type Chinese pig breed, it has the disadvantage of high carcass fatness and a lower lean rate. Compared with the DB pig, the Landrace pig, a foreign commercial pig, has the merit of faster growth, a higher lean meat percentage, and less IMF. Therefore, the two pig breeds could be compared to assess the difference in meat quality as an ideal comparison between Western commercial pigs and Chinese native pigs [18].

Accordingly, muscle is a significant factor influencing animals during the growth and development period. The growth ratio of the longissimus dorsi muscle is relatively more stable than that of other muscles. Thus, a previous study focused on and revealed mRNA or miRNA expression levels in longissimus dorsi muscle using high-throughput RNA-seq analysis of animals during growth and development periods [19,20]. On the other hand, RNA-seq is an efficient way to map and quantify the transcriptome and to analyze differentially expressed genes (DEGs) in different breeds.

Because the research of the DB pig was almost in a blank stage and transcriptome analysis of the longissimus dorsi muscle in DB pigs has not yet been performed, transcriptomic analysis was utilized, along with functional enrichment of Gene Ontology (GO) terms and Kyoto Encyclopedia of Genes and Genomes (KEGG) pathway analysis, to reveal gene expression profiles in the longissimus dorsi muscle of DB and Landrace pigs. The present study aimed to identify DEGs and metabolic pathways regulating meat quality. These results could offer further insight into the mechanisms of growth and development of swine longissimus dorsi muscle and provide useful information for genetically improving pork quality.

## Materials and methods

### Experimental materials

Three sows each from the Debao breed with the average weight of 70 kg and Landrace breed with the average weight of 90 kg were kept under the same conditions for 7 months at Guangxi Baise Paiqi Co., Ltd, China. The six selected pigs were individually raised in separate stalls from birth to 7 months of age, with free access to food, water and both breeds were fed the same conventional diet. Pigs were killed by lethal severing of the anterior inferior iliac artery, and a 20 g sample of the longissimus dorsi muscle was collected. The samples were immediately snap-frozen in liquid nitrogen and stored at −80°C until use. RNA extraction was performed in the laboratory.

All animals were administered general anesthesia (Zoletil 50, Virbac Co., France) before killing, and they did not suffer unnecessarily at any stage of the present study. All animal procedures were approved by the Committee on the Ethics of Animal Experiments of Guangxi University (Protocol Number: GXU2017-014) and were conducted in accordance with the National Research Council Guide for Care and Use of Laboratory Animals (2017). The data about it were upload to the NCBI, and the relevant accession number is PRJNA 541113.

### Total RNA extraction and mRNA purification

Total RNA was extracted from the longissimus dorsi muscles of Debao pigs and Landrace pigs according to the TRIzol method. It was hybridized with a biotinylated Oligo probe to allow the mRNA to bind to the probe. The microcentrifuge tube used in the hybridization was treated with DEPC water, and the appropriate amount of total RNA was added. RNase-free sterile water was added to 0.5 ml, the tube was placed in a preheated water bath for 60 min, and then 2 µl of biotin-labeled probe was added. The probe was hybridized with the RNA, gently mixed, and allowed to stand at room temperature for 10 min. After completion of the hybridization, SA-PMPS affinity magnetic beads were used for separation and purification, and the magnetic beads mixed in the solution were placed in a magnetic stand to separate the solution from the magnetic beads. Then, the solution was aspirated, and the beads were washed with 0.5 ml of 1× SSC solution three times and resuspended in 200 µl of 1× SSC. The RNA bound to the probe was added to the magnetic beads, gently mixed, and allowed to stand for 10 min, during which the magnetic beads were suspended. The tube was then placed in a magnetic stand for bead separation, and the beads were washed with 300 µl of 0.1× SSC. After this step was completed three times, the beads were resuspended with 200 µl DEPC, the tube was placed into a magnetic rack, and the solution was then transferred to a new tube. After cleaning, the two aqueous phases were combined, and 10% NaAC was added to the tube at the same volume. After addition of isopropanol, the tube was placed in a −20°C freezer overnight, centrifuged at 4°C, vacuum dried, and stored in a −80°C freezer for later use.

### Detection of total RNA and quality of extracted mRNA

For Agilent 2100 purity and concentration detection, 1 µl each of the extracted total RNA and mRNA were sent to Kiddio:

1. Pretest treatment (detection concentration guide interval: 25–500 ng/µl - Nanodrop value):
  - When the sample concentration Nanodrop value was within the recommended interval, it was directly detected by the Agilent 2100;
  - When the sample concentration Nanodrop value was higher than the recommended interval, the concentration was diluted to the appropriate interval for Agilent 2100 detection, and the detected value was multiplied by the dilution factor to obtain the original sample concentration;
  - When the sample concentration Nanodrop value was lower than the guide interval, the test was stopped, and the feasibility of using an Agilent 2100 RNA 6000 Pico kit was assessed.
2. Purity and concentration detection
  - Preliminary quantification - NanoDrop 2000 spectrophotometer detection:
    - NanoDrop 2000 was used to detected the blank reference, ddH_2_O.
    - Accurate quantification of concentration - Agilent 2100 RNA 6000 Nano kit detection.

### RNA-seq

#### cDNA library construction

After total RNA extraction, it was used the magnetic beads with Oligo (dT) to enrich mRNA, and then, it was added the fragmentation buffer to make it become short fragment. After that, it becomes a template to compose the first strand of cDNA by using random hexamers, and then, buffer, dNTPs, RNase H, and DNA polymerase I was added to compose the second strand of cDNA. Besides, it was needed to purify by QiaQuick kit, to washout through end repair, to add base A, and to add linker by EB buffer, and then, the target size fragment was recovered by agarose gel electrophoresis and PCR amplification was performed to complete the library construction work, which used the Illumina HiSeq™ to RNA-seq. The main kit, during this process, was NEB#7530 kit (NEB#7530, New England Biolabs).

#### cDNA library quality testing

After the library was successfully constructed according to the above steps, the main kit, in this step, was High Sensitivity DNA assay kit. It could test the sample in the range of 50–7000 bp and its concentration range was 5–500 pg/µl.

#### RNA-seq

The constructed cDNA library was subjected to high-throughput detection.

#### Access to data bioinformatics analysis

Biological analysis of the data obtained from high-throughput testing was performed as follows: first, the obtained raw data were subjected to quality control and compared with the reference genome. The quality of the transcriptome library was evaluated, and the detected genes were analyzed by gene structure analysis and gene expression analysis. Gene structure analysis mainly included single nucleotide polymorphism (SNP) analysis, variable shear sequencing, and gene structure optimization; gene expression analysis mainly included DEG screening, DEG clustering, functional annotation etc.

Then, using TopHat technology, the read segments in the extracted samples were compared with the reference genome to obtain the alignment results of the samples. The obtained transcripts were assembled, requiring Cufflinks to complete the assembly process and thereby obtaining an assembled sample. In the process of assembling the sample, the raw data underwent primary preprocessing, the remaining ribosomes were removed, and clean data were obtained. However, of paramount importance is removing the low-quality and joint data in this process. Next, for the reference genome comparison, the draft assembly was transcribed, and all the transcripts were obtained.

Finally, to obtain the target genome at different levels of expression, the samples were first grouped and combined, and then the data from different groups were combined and expressed. Both steps needed to be completed by Cuffmerge Technology. During the course of the study, if multiple samples were processed separately, the results obtained from the individual samples were processed, the excess gene sequences were removed by combining, errors occurring during assembly in a single sample were eliminated, and the remaining genes were optimized and assembled into as long of a gene sequence as possible. The obtained gene sequence and the already-defined reference gene sequence were subjected to differential expression analysis, and during the expression analysis, significantly different results were plotted and displayed. Often, biological repeats of gene sequences exist in the gene sequence. In such cases, the gene sequence was analyzed in order to identify component differences between groups, and a differential mapping cluster map was also used in this situation, which was convenient for later experiment.

#### Sequencing data quality control

To ensure standardization of the bioinformatics analysis, the obtained readings needed to be controlled for quality; unrecognized bases and low-quality reads were cleaned to obtain clean reads for subsequent analysis. The Q-score is the base quality value and a mapping to the probability of base identification error. It is directly proportional to the base quality value and the accuracy of base recognition. Through sequencing, the initial data gained were the original data images, which were then converted by base calling into sequence data, the raw reads that were stored in the FASTQ file format. Each read is depicted by four lines in a FASTQ format file:

@A80GVTABXX:4:1:2587:1979#ACAGTGAT/1
NTTTGATATGTGTGAGGACGTCTGCAGCGTCACCTTTATCGGCCATGGT+BTTMKZXUUUdddddddddddddddddddddddddddadddddd∧WYYU

The obtained sequence of each raw read has four rows of data. The first row and the third range are the names of the sequences, which are the names of the series. The third procession after the ‘+’ is omitted for saving, which is based on sequencing. The instrument determines the second line of information, which is the detected gene sequence, and the last row is a description of the quality of the detection of the gene sequence. Each character corresponds to each base in the second row. The ASCII value, which is the quality of the sequencing of the detected base minus 64, corresponds to each character in the fourth line. Base quality values ranged from 2 to 41 after processing via the Illumina GA Pipeline v1.5. Illumina HiSeq™ sequencing error rates and sequencing quality values have a concise correspondence (Table 1).

**Table 1:** A simple correspondence relationship between sequencing error rate and sequencing quality value

Sequencing error rate	Sequencing quality	Corresponding character
1%	20	T
0.1%	30	∧
0.01%	40	h

#### Sequence comparison

The transcriptome-sequenced values were compared, the obtained clean reads from DB pigs and Landrace pigs were compared and analyzed by the relevant software, and the comparative results were statistically analyzed. The reference genome originate was Ensembl release 90 database, and the version was Sus_scrofa.Sscrofa11.1.

#### Analysis of gene expression

The expression levels of transcripts and genes were analyzed, and Fragments Per Kilobase of transcript per Million mapped reads (FPKM) was used as a measure to complete this analysis by using Cufflinks software and Cuffdiff components.

### Differential gene analysis

#### Differential gene screening

Differential genes were screened according to the edgeR filter criteria (log2|fold change| > 2, FDR < 0.05). A volcano plot was used to display the screening results. The abscissa indicates log2 (fold change), and the ordinate indicates −lg (FDR). Red indicates significant up-regulation and green indicates significant down-regulation.

#### Differential gene function annotation and functional enrichment analysis (GO and KEGG)

To better understand the functions of the DEGs, we screened the related signaling pathways involved. First, the DAVID6 database with the genome of the pig as the background value was used for functional annotation of the DEGs. Second, Blast2 GO software with a value ≤le-5 was used to classify the differentially expressed data. Third, the GO function classification was analyzed and plotted, hypergeometry was assessed with WEGO software, and validation and screening were performed for genes enriched with GO significant terms. Finally, KEGG pathway enrichment analysis was performed for DEGs by statistical analysis using KOBAS online software.

#### Protein–protein interaction network analysis

To uncover the genes that play a key role, the DEGs were screened for protein–protein interaction (PPI) network analysis: on the one hand, an online database was used to determine the interaction between these genes; on the other hand, it was visualized by Cytoscape 5.0 software. Moreover, CytoNCA was used to analyze the tightness of the interactions between these genes to identify hub genes that were determined using a degree greater than or equal to 10 as the screening criterion.

#### Verification of DEGs by real-time PCR

To verify the reliability of our sequencing results, ACTB was used as the internal reference. In addition, several important genes were selected from the mRNA samples of DB pigs and Landrace pigs. Real-time PCR primers were designed using Primer3 software according to standard fluorescent PCR primer principles (Table 2).

**Table 2:** DEG verifying primer sequence

Gene symbol	Primers
*ACTB*	CTCCGATCTGTGCAGGGTAT
	TGTGAATGCAAACGCTTCCA
*FOS*	TGAACGAGTTTCGGTATGGCG
	CATTCAGGAACGAACTGATAGCA
*CCL2*	AGTGGTCAGTCCAACACTCTG
	GAGACCTCCAGGGTATCTTGAA
*IL6*	GACCACAGGATGATCCACTTAGC
	ACCTTTAGGCCCTAGCATCAC
*JUNB*	AGCCCATCCCCGCTGTCCATAAAG
	CAGGGTCAACTGTACAGGCATCTT
*GATA6*	CACAGTTCTCAAAGCACAGCG
	GGACGGTAACGGGAATGTATG
*ANKRD1*	CCAAATCCACGCTTGTGTTGA
	GGAATGAGTAGACCTCCACCT

## Results

### Sequencing quality assessment

#### Sequencing raw read quality assessment

**Figure 1:**
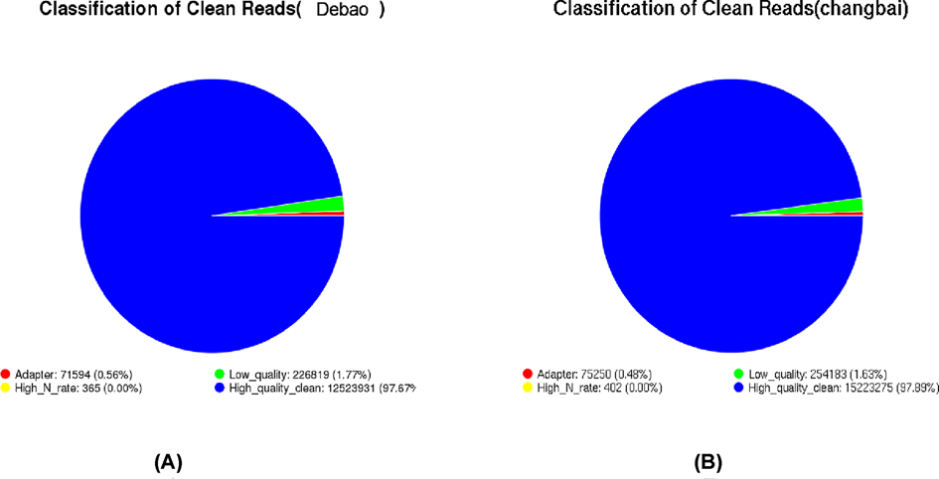
Debao pig and Landrace samples classification of clean reads (**A**) Samples of Debao pig classification of clean reads; (**B**) samples of Landrace classification of clean reads.

By analyzing the quality of the original sequencing reads, the distribution of the proportions of various reads in the sample test was revealed. DB pig sample clean reads contained 0.56% of the linker sequence, the number of unknown base reads was 0.00% of the total number of reads, the number of low-quality reads accounted for 1.77% of the total number of reads, and the data obtained by removing the impurities from the original sequence data accounted for 97.67% of the total number of reads. The number of reads containing the linker sequence in the clean reads of the Landrace sample accounted for 0.48% of the total number of reads, the number of unknown base reads was 0.00% of the total number of reads, the number of low-quality reads was 1.63% of the total number of reads, and the data obtained by removing the impurities from the original sequence data accounted for 89% of total reads (Figure 1).

#### Base composition and base mass analysis

**Figure 2:**
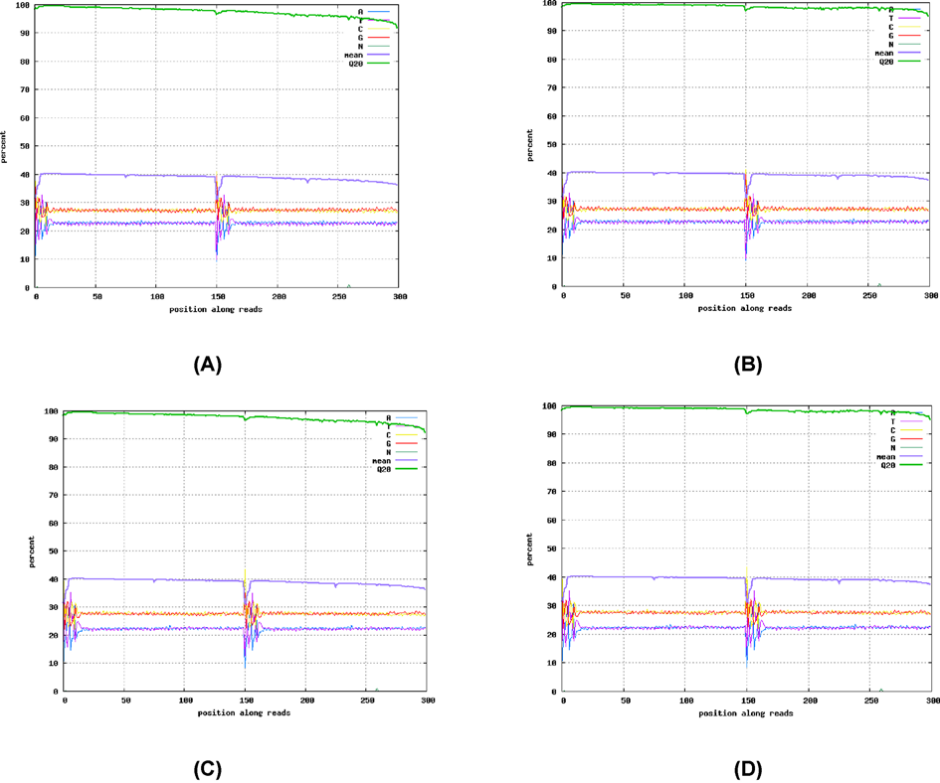
Samples of base composition distribution (**A**) Samples of Debao pig before filtration base composition distribution; (**B**) samples of Debao pig after filtration base composition distribution; (**C**) samples of Landrace before filtration base composition distribution; (**D**) samples of Landrace after filtration base composition distribution.

Base composition and mass value distribution diagrams were generated for the prefiltered and filtered data to illustrate the data quality. According to the distribution map, both the DB pig and Landrace pig had a GC content higher than the AT content. In other words, the GC had a specific gravity of more than 50%. The bases with a mass value ≥ 20 were increased in the filtered reads at each position. On the x-axis, 1–125 bp represented the base position of read1 and 126–250 bp represented the base position of read2. The A, T, C, and G curves each represented the ratio of bases A, T, C, and G at each position. If the base composition in the detected genome did not break the equilibrium, the obtained curves must be bases A and T. The curve was completed, and the curves of base G and base C coincided. Otherwise, a mismatch occurred. In the base analysis, if the base ratio at a certain position was not detected, it was represented by an N curve. The average mass of a base at each position referred to the average mass of all bases and was represented by a mean curve. The Q20 curve represents the base ratio of the bases at each position with a mass value ≥ Q20 (Figure 2).

### The results of alignment statistics

#### Alignment of ribosome results

The statistical comparison between HQ clean data and ribosomal RNA was shown as follows: the number of all reads in Debao pig samples was 25047862, the number of rRNAs in the comparison was 144838, the proportion of the total was 0.58%, and the number of reads without rRNA was 24030324, accounting for 99.42% of the total. The total number of reads of Landrace pig samples was 30446550, the number of rRNAs was 217142, the proportion of the total was 0.71%, and the number of reads without rRNA was 30229408, accounting for 99.29% of the total (Table 3).

**Table 3:** HQ clean data and rRNA comparison statistics

Sample	All Reads Num	Mapped reads	Unmapped reads
Debao	25047862	144838 (0.58%)	24903024 (99.42%)
changbai	30446550	217142 (0.71%)	30229408 (99.29%)

Sample, sample name; All Reads Num, total reads.

Mapped Reads: Compared the number of reads of rRNA and the proportion of the total.

Unmapped Reads: No comparison of the number of reads of rRNA and the proportion of the total.

#### Alignment of reference genome results

TopHat was used to compare DB pigs and Landrace pigs with reference genes. The total number of reads measured in DB pigs was 24030024; the number of reads on the unreferenced reference genome was 2284728, accounting for 9.17% of the total, and the number of reads only on the reference genome was 22215676, accounting for 89.21% of the total. Furthermore, the number of reads of the reference genome in multiple comparisons was 402620, accounting for 1.62% of the total, and the mapping ratio was 90.83%. The sum of reads measured in Landrace pigs was 30229408; the number of reads on the unreferenced reference genome was 2098698, accounting for 6.94% of the total, and the number of reads only on the reference genome was 27570788, accounting for 91.21% of the total. Moreover, the number of reads of the reference genome in multiple comparisons was 559922, accounting for 1.85% of the total, and the mapping ratio was 93.06% (Table 4).

**Table 4:** Comparison of Unmapped Reads with reference genome after ribosome comparison

Sample	Total reads	Unmapped reads	Unique mapped reads	Multiple mapped reads	Mapping ratio
Debao	24903024	2284728	22215676	402620	90.83%
changbai	30229408	2098698	27570788	559922	93.06%

Sample, the name of sample; Total reads, total number of reads.

Unmapped Reads: Unmatched reads of the reference genome and the proportion of the total.

Unique Mapped Reads: The number of reads in the unique reference genome and the proportion of the total.

Multiple Mapped Reads: The number of reads of the reference genome in multiple comparisons and the proportion of the total.

#### Sequencing saturation analysis

**Figure 3:**
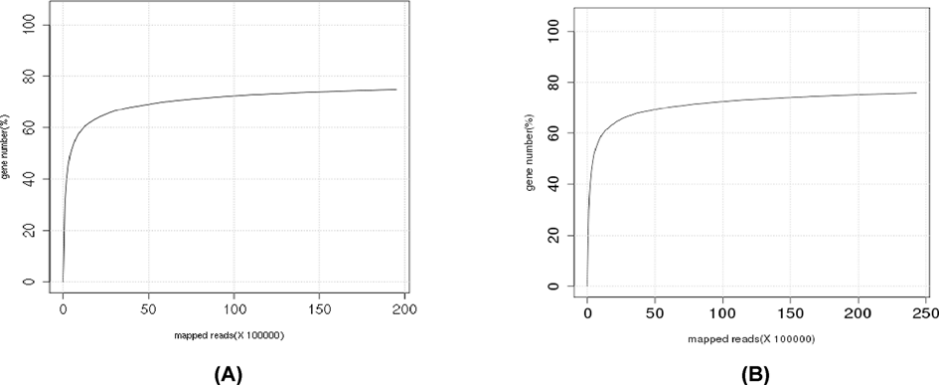
Samples of sequencing saturation analysis (**A**) Samples of Debao pig sequencing saturation analysis; (**B**) samples of Landrace sequencing saturation analysis.

When sequencing the number of genes, if saturation is not considered, the detected gene value is directly proportional to the level of detection. Actually, as the level of detection increases, when a certain value is reached, the detected gene value will no longer increase and tends to balance; this point is the saturation of the test, and sequencing saturation analysis is a measure of whether a sample’s sequencing level is saturated. As seen from the figure, when the number of DB pig and Landrace pig sequences reached 5 million, the results were balanced, indicating that the number of genes detected reached 5 million and that the genes became saturated (Figure 3).

#### Sequencing randomization analysis

**Figure 4:**
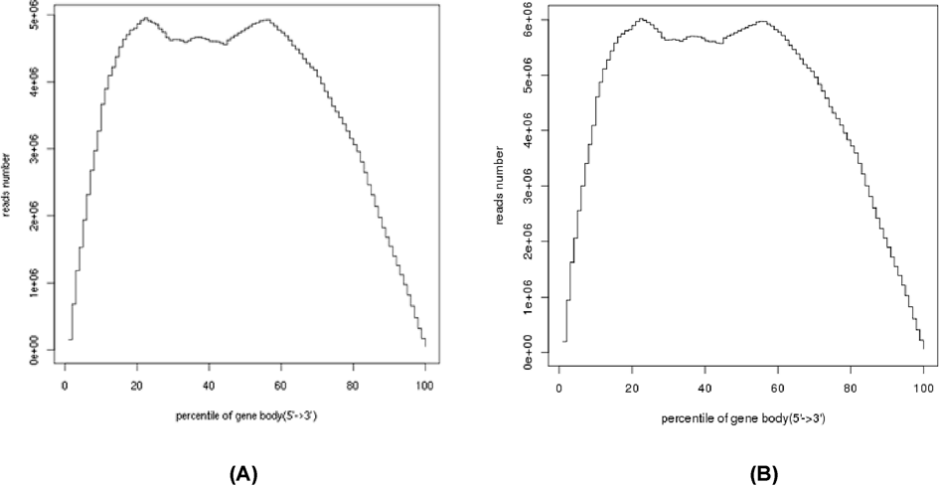
Analysis of random samples (**A**) Analysis of random samples of Debao pig; (**B**) analysis of random samples of Landrace.

The degree of random interruption of mRNA was determined by the distribution of the reads on the reference gene, but the length of the reference gene varied depending on the reference gene. However, the value of the reads obtained after the reading at the normalized position of the reference gene was the ratio of the length of the read gene to the position of the gene. By reading the position of the segment, different readings corresponding to different positions on the gene were determined and counted. The more uniform the distribution should be in all parts of the gene, the better the randomness is interrupted. The randomness of the 5′–3′ gene of DB pigs and Landrace pigs in the figure indicates that the random distribution of the two samples was more uniform (Figure 4).

### Gene statistics

#### The results of gene expression statistics

The table shows that the total number of genes detected in all samples was 22342 for the reference genome; the number of known genes detected was 14743, accounting for 65.99% of the total number of genes in the reference genome; and the number of new genes was 190 (Table 5). From the table, the statistics of the number of genes in DB pig and Landrace pig samples showed that the number of known genes detected in DB pig samples was 13994, accounting for 62.64% of the number of genes in the reference genome; the number of new genes detected was 174; and the number of all genes was 14168. In addition, the number of known genes detected in Landrace pig samples was 14223, accounting for 63.66% of the number of genes in the reference genome; the number of new genes detected was 181; and the number of all genes was 14404 (Table 6).

**Table 5:** Statistics of the number of samples detected in all samples

All Reference Genes	Known Gene Num	New Gene Num
22342	14743 (65.99%)	190

All Reference Genes: total number of genes in the reference genome; Known Gene Num: Number of known genes detected (ratio = number of known genes/total number of genes in the reference genome); New Gene Num: number of new genes detected.

**Table 6:** Statistics of the number of samples detected in each sample

Sample name	New Gene Num	All Gene Num
Debao	174	14168
changbai	181	14404

#### Gene coverage statistics

**Figure 5:**
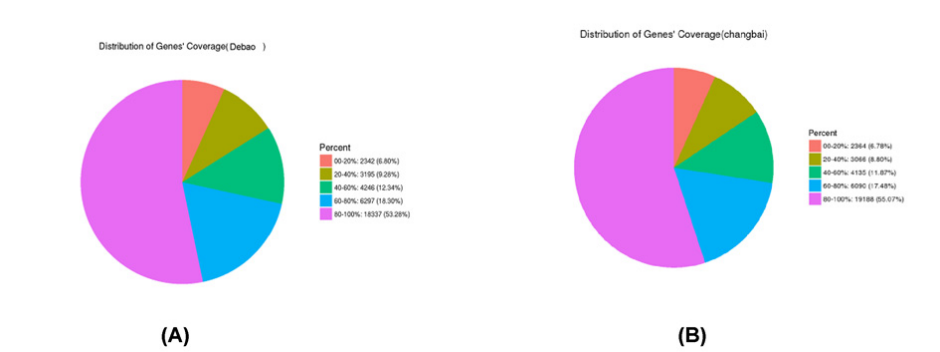
Gene coverage statistics (**A**) Debao pig gene coverage statistics; (**B**) Landrace gene coverage statistics.

The percentage of reads in the genetic test that are located in genes is gene coverage, which is the ratio of the number of bases in the read to the total number of bases of the gene located in the read. The figure shows that there were 18337 reads with a read gene coverage of more than 80% detected in DB pig samples, accounting for 53.28% of the total reads; the number of reads with a coverage of 60–80% was 6297, accounting for 18.30% of the total reads; the number of reads with a coverage of 40–60% was 4246, accounting for 12.34% of the total reads; the number of reads with a coverage of 20–40% was 3195, accounting for 9.28% of the total reads; and the number of reads with a coverage below 20% was 2342, accounting for 6.8% of the total. In the Landrace pig sample, 19188 reads had more than 80% read coverage, accounting for 55.07% of the total; the number of reads with a coverage of 60–80% was 6090, accounting for 17.48% of the total reads; the number of reads with a coverage of 40–60% was 4135, accounting for 11.87% of the total; the number of reads with a coverage of 20–40% was 3066, accounting for 8.80% of the total; and the number of reads with a coverage less than 20% was 2364, accounting for 6.78% of the total. Therefore, the read coverage of the detection between the two breeds was similar (Figure 5).

#### Expression statistics

The Cufflinks method was used to calculate the gene expression of the target gene, and the gene expression of all the genes in the samples to be tested was calculated (Table 7).

**Table 7:** The expression quantity and annotation summary of all genes (only show the expression of the first sample of the first ten lines)

id	Bebao_fpkm	……	Debao_count	……	Symbol	Description	KEGG_A_class	KEGG_B_class	Pathway	K_ID	GO component	GO function	GO process
ENSSSCG00000000002	0.49	……	11.00	……	GTSE1	PREDICTED…	Cellular…	Cell grow…	ko04115//…	K10129	-	GO:000548…	GO:004476…
ENSSSCG00000000003	0.70	……	33.00	……	TTC38	PREDICTED…	-	-	-	-	-	-	-
ENSSSCG00000000005	3.08	……	26.00	……	CDPF1	PREDICTED…	-	-	-	-	-	-	-
ENSSSCG00000000006	2.91	……	40.00	……	PPARA	peroxisom…	Organisma…	Endocrine…	ko04024//…	K07294	GO:004322…	GO:000107…	GO:004470…
ENSSSCG00000000007	7.21	……	108.00	……	TRMU	PREDICTED…	Genetic I…	Folding…	ko04122//…	K00566	GO:004442…	GO:001674…	GO:000639…
ENSSSCG00000000010	14.08	……	568.00	……	FBLN1	PREDICTED…	-	-	-	-	GO:000557…	GO:009877…	GO:004476…
ENSSSCG00000000014	6.70	……	160.00	……	FAM118A	PREDICTED…	-	-	-	-	-	-	-
ENSSSCG00000000018	7.55	……	329.00	……	KIAA0930	PREDICTED…	-	-	-	-	-	-	-
ENSSSCG00000000019	11.45	……	611.00	……	NUP50	PREDICTED…	Genetic I…	Translati…	ko03013//…	K14295	GO:003196…	-	GO:005117…
ENSSSCG00000000020	0.50	……	7.00	……	PHF21B	PREDICTED…	-	-	-	-	-	GO:004316…	-

#### Expression abundance distribution

**Figure 6:**
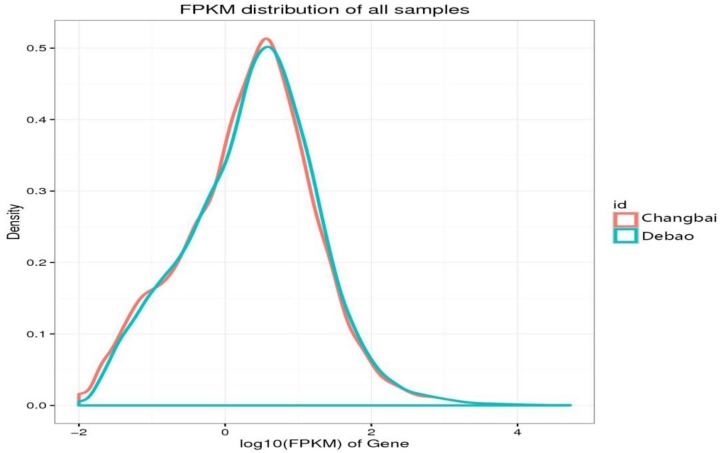
Debao and Landrace pigs expression abundance distribution The red line is the expression abundance distribution of the Changbai pig samples, and the green line is the expression abundance distribution of the Debao pig samples.

The expression levels of the detected genes were calculated by the Cufflinks method, and the gene expression density distribution plot was prepared by analyzing the relationship between the log_10_(FPKM) and the density of the gene expression. The larger the abscissa value was, the larger the expression level of the gene, and the numerical value of the ordinate was the ratio of the level of gene expression relative to the total number of genes that had been detected; in other words, it was the gene expression density. A curve in the coordinates represented a sample, and as the expression level gradually increased, the peak was the region where gene expression was most concentrated. By comparing the expression density of the longissimus dorsi muscle samples from Debao pig and Landrace pig by coordinates, the most concentrated areas of gene expression in Debao pigs and Landrace pigs were the same, and the value of the concentrated area of gene expression in Landrace pigs found on the plot was higher. Landrace pigs had higher gene expression density and higher gene expression in the most concentrated regions (Figure 6).

#### New gene annotation

The reliable screening conditions for new genes were: length ≥200 bp and exon number ≥ 2 in this experiment. By analyzing the detected gene transcripts and comparing them with known genomes, new gene transcripts were detected in the genome and then annotated (Table 8).

**Table 8:** New gene annotation table for all samples (only the first ten lines are shown)

GeneID	Symbol	Description	KEGG_A_class	KEGG_B_class	Pathway	K_ID	GO component	GO function	GO process
XLOC 000093	-	-	-	-	-	-	-	-	-
XLOC 000137	-	-	-	-	-	-	-	-	-
XLOC 000361	-	-	-	-	-	-	-	-	-
XLOC 000456	-	-	-	-	-	-	-	-	-
XLOC 000610	-	-	-	-	-	-	-	-	-
XLOC 000731	FAM206A	PREDICTED:prote	-	-	-	-	-	-	GO:0044281//smal
XLOC 000869	-	PREDICTED:uncha	-	-	-	-	-	-	-
XLOC 000878	-	-	-	-	-	-	-	-	-
XLOC 000928	-	-	-	-	-	-	-	-	-
XLOC 000961	-	-	-	-	-	-	-	-	-

### Difference analysis

#### Sample relationship overview

**Figure 7:**
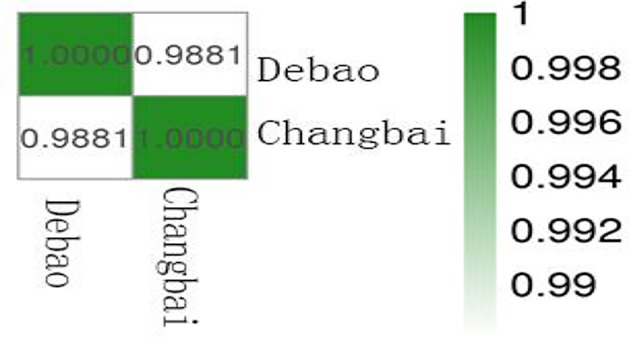
Debao and Landrace pigs correlation chart The abscissa and the ordinate were the respective samples, and the abscissa and the ordinate of each patch represented the correlation between the Debao pig samples and the Changbai pig samples. Importantly, two completely related genomes had a value of 1. Closer to 1 the relative value was, the larger the Pearson correlation coefficient for the Debao pig samples and the Changbai pig samples; conversely, closer to 0 the relative value was, the smaller the Pearson correlation coefficient between the Debao pig samples and the Changbai pig samples.

To generate a correlation chart, the Pierce correlation coefficient between the expression levels of each gene (the entire gene set) in any two samples was calculated, and then these correlation coefficients were visually displayed as a chart between the two samples. In the figure, the abscissa and the ordinate were the respective samples, and the abscissa and the ordinate of each patch represented the correlation between the X sample and the Y sample. Importantly, two completely related genomes had a value of 1. Closer to 1 the relative value was, the larger the Pearson correlation coefficient for the X sample and the Y sample; conversely, closer to 0 the relative value was, the smaller the Pearson correlation coefficient between the X sample and the Y sample. The correlation coefficient of the longissimus muscle from Debao pig and Landrace pig was 0.9881, indicating that the Pearson correlation coefficient of the two samples was very large (Figure 7).

### Genomic structure analysis

#### Genomic optimization

**Figure 8:**
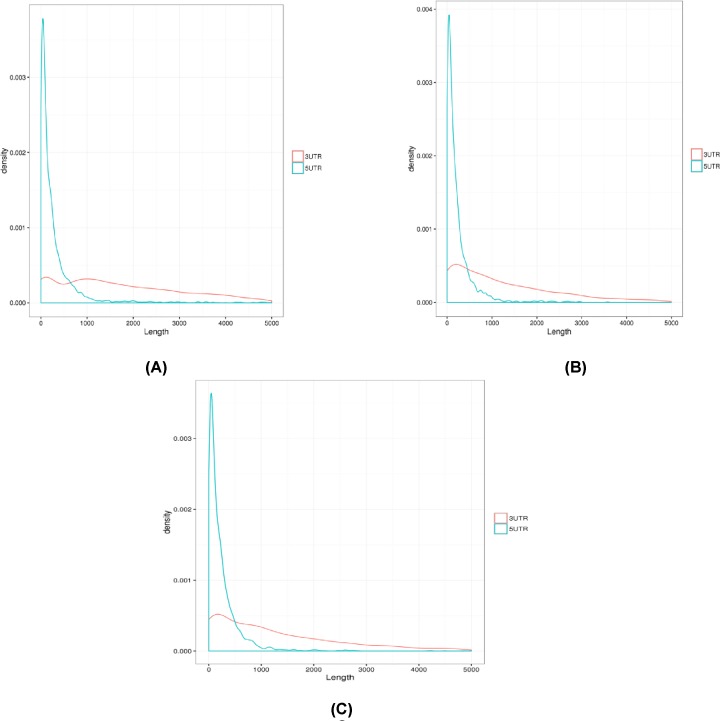
Samples of structure diagram (**A**) All samples of genetic structure optimization; (**B**) the structure diagram of Debao pig samples; (**C**) the structure diagram of Landrace samples.

The development of biology began around biological models such as humans and mice, so the gene annotation for these species is more thorough; however, for other species, gene annotation was relatively lacking, and the genome structure can be assessed by analyzing the sequencing reads. Gene annotation information for these species needed to be optimized; thus, refining new gene annotation information for these species was necessary. On the one hand, reverse transcription of the reads was performed, and the obtained reverse transcript was reconstructed; on the other hand, sequences with known reference transcripts were compared by Cufflinks to detect recombination that might increase the gene annotation in the new reverse transcript. Therefore, the optimization of the genome structure and improvement of gene annotation information were completed for specific species (Figure 8).

#### Alternative splicing analysis

**Figure 9:**
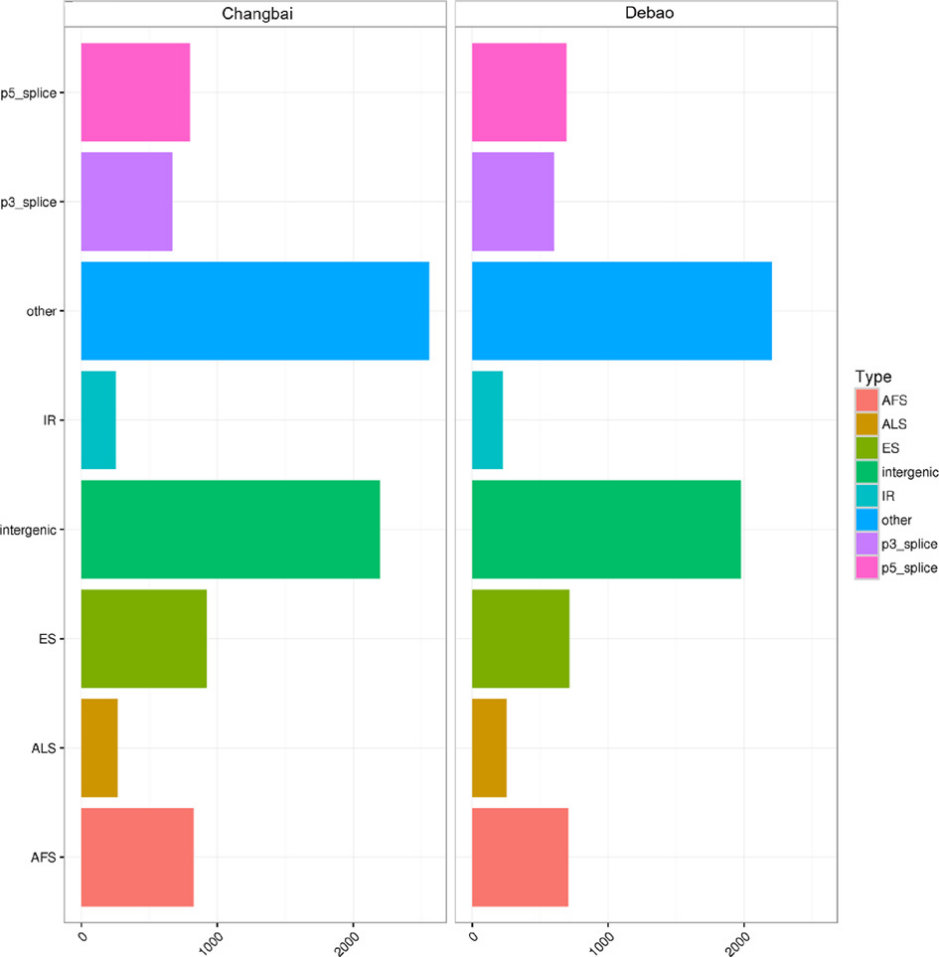
Analysis of variable shear events The vertical axis is the abbreviation of the variable shear event, and the horizontal axis is the number of variable shears under the event. Different samples are distinguished by different subgraphs and colors.

Alternative splicing exists in eukaryotic clocks because of the presence of introns and exons in the genomes of eukaryotes, requiring cleavage and ligation during transcription. After transcription, pre-mRNA is spliced by the action of the relevant enzyme to form mRNAs with different biological functions and then translated into proteins with different biological functions. The same gene might encode different proteins because of different methods of cleavage, so a protein encoded by a gene may not be the only one. Among the many reasons for the diversity of proteins, variable shear is extremely important. Alternative analysis of Landrace pig and DB pig was important for assessing the biological function of the longissimus dorsi muscle in the back. All splicing site data from the TopHat comparison results were filtered to include only those data with no less than five reads in order to avoid results due to error. Furthermore, the filtered data were compared with known shear sites (allowing 1 bp error). At last, the known shear sites were detected, and the remaining new shear sites by variable shear events were counted and classified. In the figure, the vertical axis is the abbreviation of the variable shear event, and the horizontal axis is the number of variable shears under the event. Different samples are distinguished by different subgraphs and colors (Figure 9).

#### Differential gene expression analysis between samples

The analysis of DEGs between samples was mainly assessed through screening differential gene expression analysis, differential gene expression pattern cluster analysis, DEG functional enrichment analysis, and DEG PPI network analysis.

#### Differential gene expression screening between samples

**Figure 10:**
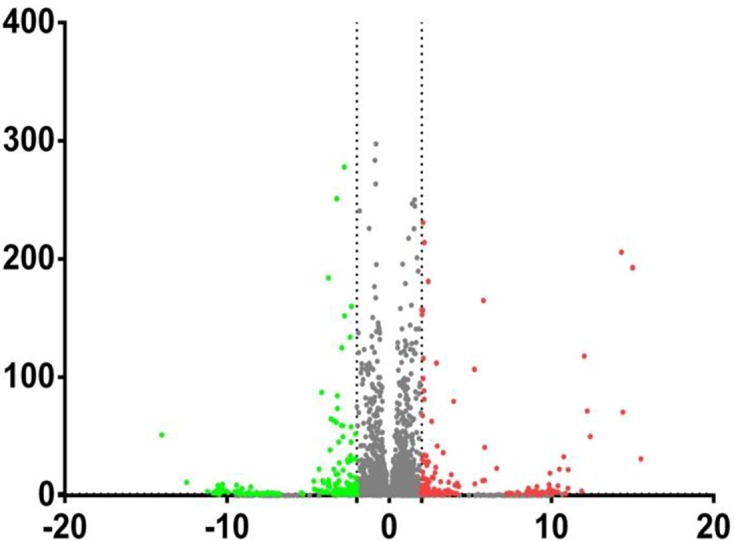
Debao and Landrace pigs samples of gene expression differences between volcano map The abscissa indicates log2FC, the ordinate indicated −lg(FDR), red dots indicate differential expression of up-regulated genes, green indicates differential expression of down-regulated genes, and gray indicates no differential expression.

A significant analysis of the differences in RNA-seq data was performed by analyzing the significance of differences between groups using the R language software package (edgeR). FDR and log2FC were used to screen differential genes with FDR < 0.05 and |log2FC| > 2. The results of the difference analysis between the two samples were displayed using a volcano plot. The abscissa indicates log2FC, the ordinate indicated −lg (FDR), red dots indicate differential expression of up-regulated genes, green indicates differential expression of down-regulated genes, and gray indicates no differential expression. There were 410 DEGs between Debao and Landrace pigs, of which 184 DEGs were significantly up-regulated and 226 DEGs were significantly down-regulated (Figure 10).

#### Differential expression gene cluster analysis

**Figure 11:**
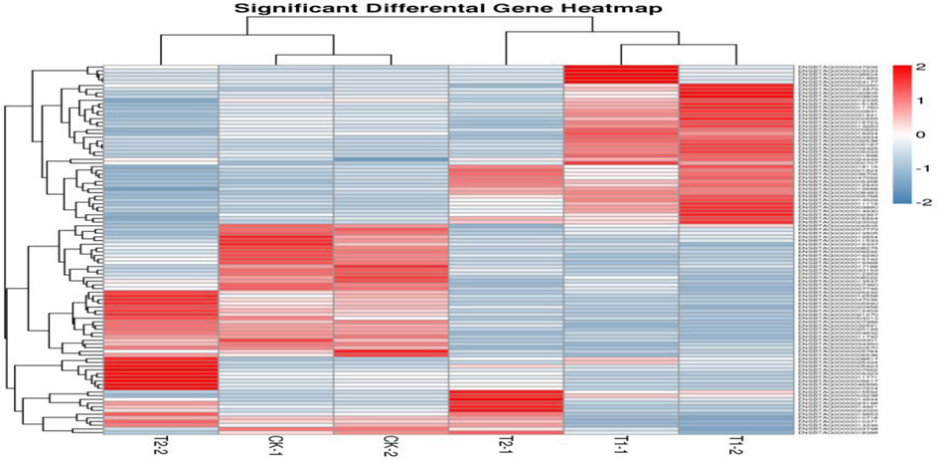
The analysis results of DB and Landrace pigs gene cluster Each data point corresponded to one row in each graph, and the intensity of the color was used to indicate the level of gene expression. The higher the expression was, the more red and dark the color; the darker the blue was, the lower the expression.

Through the analysis of gene expression and differential gene expression, the gene clustering results were more intuitively illustrated in the form of heat maps to complete the clustering of the DEGs. The gene expression in each sample was calculated by using 2 as the base, and then the cluster analysis of the detected genes was completed. In the figure, each data point corresponded to one row in each graph, and the intensity of the color was used to indicate the level of gene expression. The higher the expression was, the more red and dark the color; the darker the blue was, the lower the expression. The results of the gene expression pattern cluster analysis of all samples showed that the clustering relationship between the same breeds of pigs was close, indicating that the samples were reproducible, and that most of the gene expression patterns between the two breeds were very different. The opposite trend was also observed—some genes were highly expressed in DB pigs, while expression was low in Landrace pigs, and *vice versa* (Figure 11).

#### Functional enrichment analysis of DEGs

**Figure 12:**
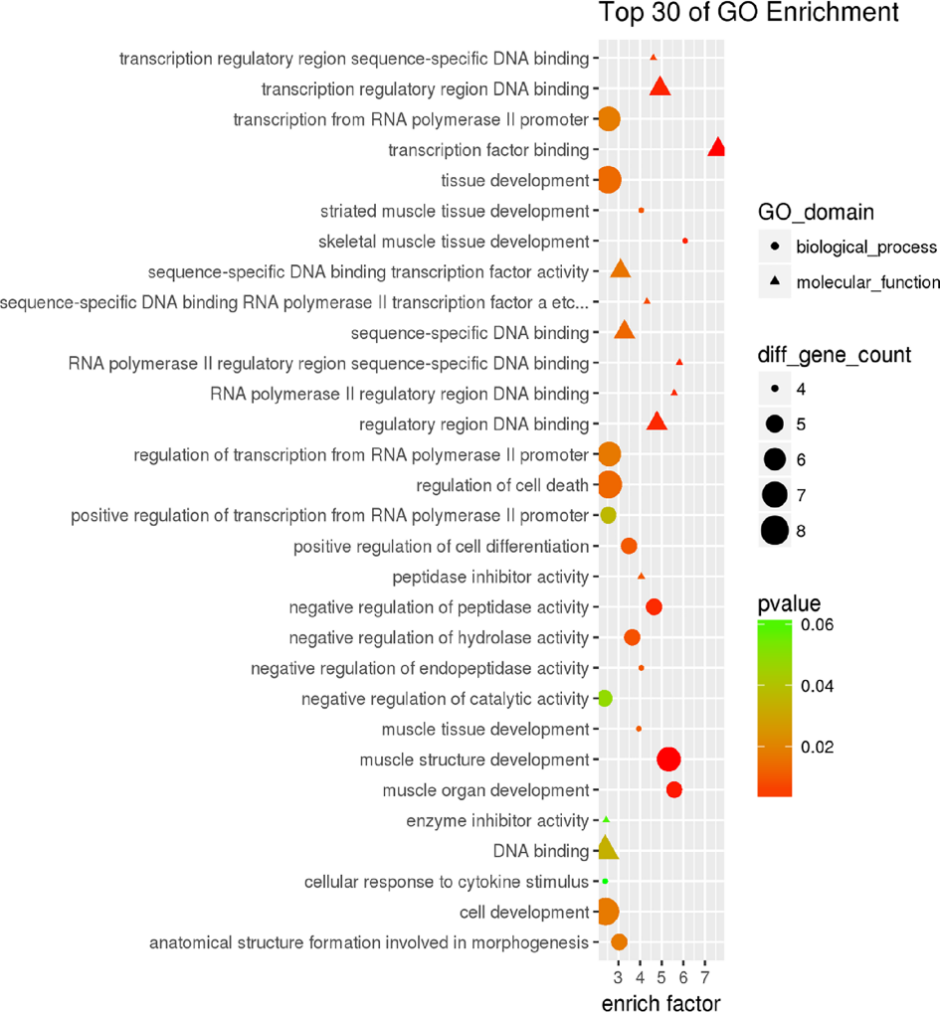
GO analysis of DEGs in Debao pig and Landrace pig The 410 DEGs were enriched in 105 GO terms because a gene often had multiple different functions; thus, the same gene would appear under different classification entries.

To better understand the function of the selected DEGs and the relationship with the phenotype, functional analysis, including GO and KEGG signaling pathway analysis, of 410 screened DEGs was performed. GO is an internationally used analysis system for classifying gene function. The analysis of gene function in organisms is mainly achieved through a continuously updated vocabulary. GO term classification statistics were performed on DEGs. Including molecular function, cellular component, and biological process, the 410 DEGs were enriched in 105 GO terms because a gene often had multiple different functions; thus, the same gene would appear under different classification entries. Thirty of them are shown (Figure 12).

**Figure 13:**
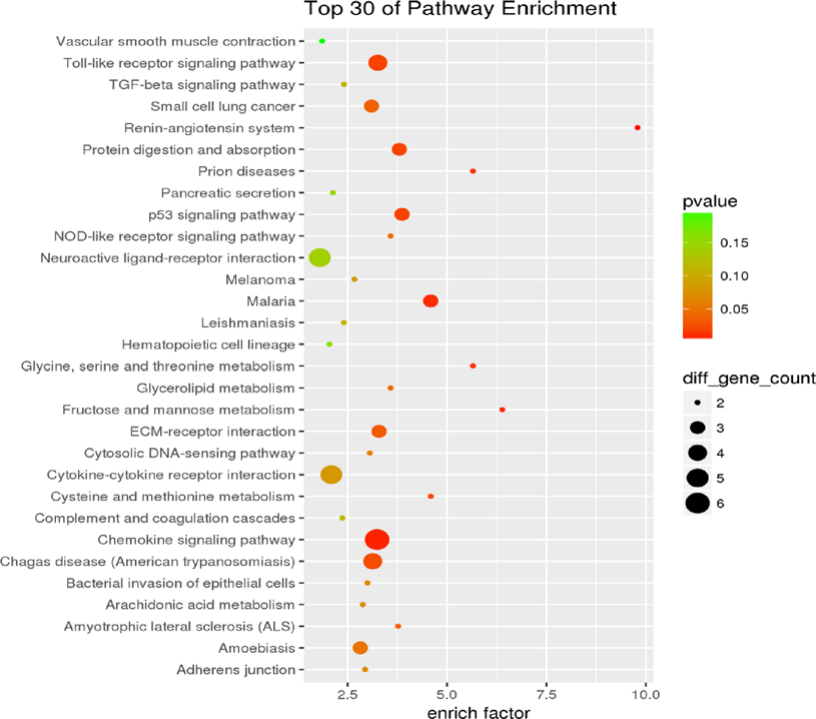
Debao and Landrace pigs KEGG gene signal pathway enrichment analysis One gene could participate in multiple KEGG signaling pathways, and these genes were discovered to participate in 94 KEGG signaling pathways.

Gene regulation in organisms was not performed by a single gene but rather by the coordination of multiple genes or products. To understand the differential gene expression of the longissimus dorsi muscle in Landrace pig and DB pig, KEGG signal pathway enrichment analysis was performed on these DEGs. One gene could participate in multiple KEGG signaling pathways, and these genes were discovered to participate in 94 KEGG signaling pathways. The first 30 signaling pathways are demonstrated (Figure 13).

#### PPI network analysis

**Figure 14:**
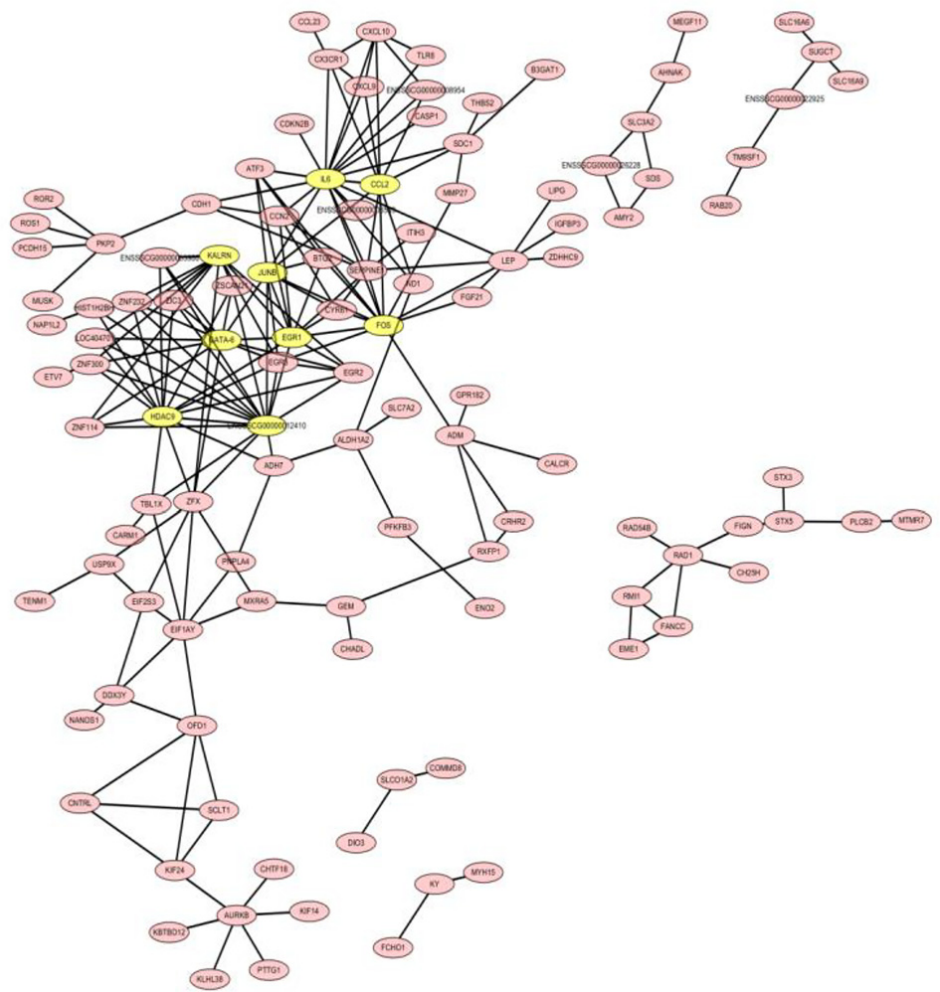
Network analysis of protein and protein interaction The 366 genes with known gene names among the DEGs were screened to identify the relationships between these genes through the STRING database; and then using Cytoscape software for visual analysis, a total of 110 nodes and 230 interactions were obtained. CytoNCA was used to calculate the degree between these genes.

To better understand interactions between the selected DEGs, several genes that played a key role in these DEGs were found, and PPI network analysis was performed on the DEGs. On the one hand, 366 genes with known gene names among the DEGs were screened to identify the relationships between these genes through the STRING database; on the other hand, using Cytoscape software for visual analysis, a total of 110 nodes and 230 interactions were obtained. CytoNCA was used to calculate the degree between these genes. Several genes with a key role, such as CCL2, IL6, JUNB, KALRN, FOS, and EGR1, were found by setting the degree value greater than 10. Yellow indicates a key gene with a degree greater than 10 (Figure 14). Some of the key genes screened, such as IL6, FOS, and EGR1, were mainly involved in metabolism, muscle development etc., indicating that these screened key genes might play an important role in phenotypic differences (meat quality or growth cycle) between the two pig breeds.

#### Real-time quantitative PCR verification of DEGs

**Figure 15:**
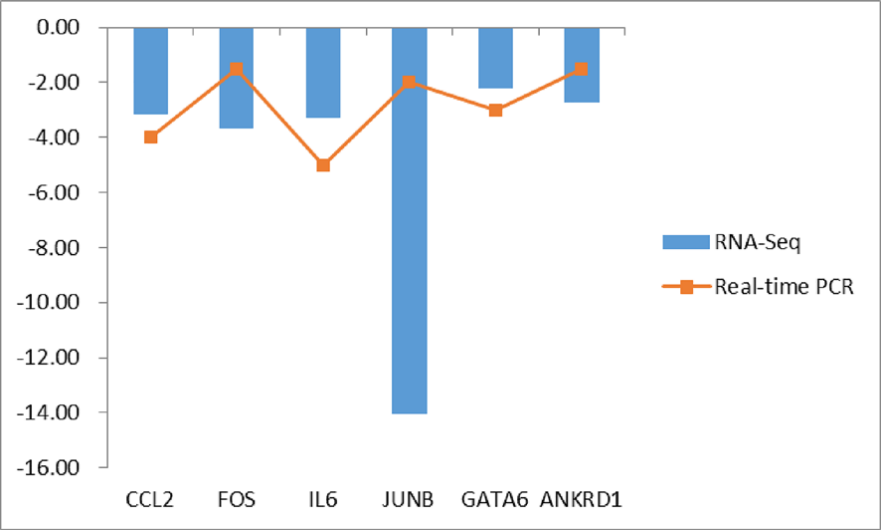
Differential expression gene real-time PCR verification The mRNA expression levels from sequencing and real-time PCR results in Debao pigs were analyzed for differential fold change relative to those in Landrace, and log2 transformation was performed.

To verify the accuracy of the RNA-seq data, six important DEGs were screened, and the expression of these genes in the longissimus dorsi muscle of DB pigs and Landrace pigs was verified by real-time PCR. The mRNA expression levels from sequencing and real-time PCR results in DB pigs were analyzed for differential fold change relative to those in Landrace pigs, and log2 transformation was performed. The results of the six genes in the real-time PCR and RNA-seq data were consistent, which explained the reliability of our RNA-seq data (Figure 15).

## Discussion

Pigs are indispensable for human life, especially in China, where the population clearly depends on pork and pig is the main agricultural economic animal [21–24]. However, with the rapid development of China’s economy, people’s living standards have greatly improved, the requirements for pork are becoming increasingly higher, and the demand for meat taste could not be satisfied with the current commercial pork [25]. The improvement in the taste of pork was mainly related to the fat content in the muscles of pigs [26]. Although the feed rate of pigs cultivated abroad was high, the growth cycle was short, but low IMF content caused the taste to be poor, which caused people to find the taste less palatable. Landrace pig is a typical lean pig, whereas DB pigs are a unique fat pig in China. However, their feed conversion rate is low, and the long growth cycle cannot meet China’s basic national conditions [27–29]. These two pig breeds, which differ greatly in phenotype, are good animal models for identifying DEGs in muscle and fat. Complex traits are phenotypes that interact due to multiple factors. Many studies have shown a significant correlation between the expression of certain genes in muscle and these complex traits [30,31]. Therefore, genomic study of the two longissimus dorsi muscle samples showed that the key genes and influencing factors causing the difference in phenotype between the two pigs could provide a basis for future genetic breeding to meet social needs [32]. In this study, the longissimus dorsi muscle of DB pigs and Landrace pigs was selected as the research sample. Total RNA was extracted, and the mRNA was purified to meet the experimental requirements. Then, the cDNA library was successfully constructed and sequenced [33–35].

Although high-throughput sequencing technology has been widely used, there are still some sequencing errors that are affected by many factors, such as the sequencer itself, sequencing reagents, and samples [36–38]. At present, the researchers found that in RNA-seq, the longer the sequence, the higher the probability of recognition errors. In general, the first few bases were prone to recognition errors during sequencing, and these few bases were just the size of the random primers during the sequencing process. Thus, the recognition errors in the first few bases were speculated to be caused by incomplete pairing of random primers with RNA templates [39–41]. Therefore, quality control of the data generated by sequencing must be performed to obtain high-quality sequencing data for subsequent bioinformatics analysis. In this regard, strict quality control was carried out on the sequencing data, and the target reads of DB pig and Landrace pig samples before and after decontamination accounted for more than 95% of the genes [42]. In the analysis of base composition and quality, GC accounts for a higher proportion than AT, with GC accounting for more than 50%, and the proportion of base mass at each position Q20 increased after denoising. In addition, through the ribosome comparison, the percentages of the rRNA in the DB pig and the Landrace pig samples were 99.42 and 99.29%, respectively [43]. Compared with the reference genome, the percentages of mapped reads from DB pig and Landrace pig longissimus dorsi muscle samples were 90.83 and 93.06%, respectively. Sequencing saturation analysis indicated that the two samples were saturated when they reached 5 million sequencing genes; the random distribution further confirmed that the reads were more evenly distributed in the genes [44]. In comparing the gene expression, gene coverage, expression and density results, the correlation coefficient of the data from the longissimus dorsi muscle of DB pig and Landrace pig was 0.9881. These data indicated that the higher quality of our sequencing data could be used for the next bioinformatics analysis. In the present study, the functional genes of DB pigs provided experimental data through the annotation of new genes in the longissimus dorsi muscle transcriptome of DB pigs and Landrace pigs, analysis of SNPs and analysis of genomic structure. More importantly, many DEGs were screened in this study [45–48].

In recent years, researchers have sequenced the longissimus dorsi muscle of warthogs and Duroc pigs and screened DEGs in the longissimus dorsi muscle of warthogs and Duroc pigs by bioinformatics analysis, obtaining 589 up-regulated genes and 364 down-regulated genes [49]. Li et al. [50] used RNA-seq technology to screen DEGs in the longissimus dorsi of Longanhua pigs and DB pigs. A total of 347 DEGs were screened, including 94 up-regulated genes and 253 down-regulated genes [50]. In this study, 410 DEGs were screened by analyzing the gene expression of DB pigs and Landrace pigs, and of these genes, 184 were significantly up-regulated, and 226 were significantly down-regulated. Furthermore, by analyzing the GO and KEGG signaling pathways of the selected DEGs, important functional genes and signaling pathways related to the difference in phenotype between DB pigs and Landrace pigs were found. A large proportion of the DEGs that had been screened were involved in metabolic regulation and muscle development.

Metabolic regulation and muscle development played an important role in pig growth and phenotype [51–53], and researchers have investigated longissimus dorsi muscles from Northeastern pigs (fat pigs) and LW pigs (lean pigs). Some studies have found differential expression of some metabolic genes, such as SCD and FAS [54]. In this study, 14 DEGs including *B3GAT1, PTGS2, AMY2, DNMT3B, PHGDH, GALNT15, PLCB2, B4GALNT1, CBR2, MBOAT1, SDS, UROC1, LIPG*, and *GLUL* were found to be involved in metabolic signaling pathways. Genes that played a key role in these DEGs, such as *CCL2, FOS, IL6, JUNB, EGR1*, and *GATA-6*, were also discovered through PPI network analysis. Through GO analysis, FOS and EGR1 were found to be involved in muscle development. In addition, studies have reported that FOS might be related to the muscle growth weight of pigs [55]. *EGR1*, an early growth response protein, plays an important role in many biological processes, such as cell proliferation, differentiation, and apoptosis. Mouse Egr1 promoted the differentiation of mouse myoblast *C2C12 in vitro* and affected cell muscle, tube fusion rate and muscle differentiation markers molecular myogenin and myosin heavy chain 2 [56]. In addition, researchers also found that the pig *EGR1* gene might be closely related to intramuscular fat content through RNA-seq technology [57]. The findings in our study that were similar to those of previous studies illustrated the reliability of our bioinformatics analysis to some extent.

Finally, some important genes were verified by real-time PCR, and these results were consistent with our sequencing results. In-depth verification of these important functional genes in the future may reveal important genes that affect pig growth and pork quality and provide a theoretical basis for future genetic breeding of new varieties that meet social needs [58,59].

## Conclusions

By sequencing the longest muscle transcriptome of DB pigs and Landrace pigs and using strict data quality control, we obtained high-quality sequencing data. By comparing the ribosomes of DB pig and Landrace pig with the reference genome, the mapping ratios of the two pig breeds were 90.83 and 93.06%, respectively. Furthermore, the sequencing saturation of the sequenced genes in the two genomes was 5 million. The random analysis of sequencing showed that the gene reads were evenly distributed in the two genomes. The number of all genes detected in DB pigs was 14168, the number of known genes was 13994, and the number of new genes was 174; the number of all genes detected in Landrace pigs was 14404, the number of known genes was 14223, and the number of new genes was 181. Gene coverage and the difference in gene expression levels of the two pig breeds were assessed by analyzing the gene expression density distribution. Analysis of the main components of data revealed significant differences between the two pig breeds. Through screening conditions of FDR < 0.05 and |log2FC| > 2, a total of 410 DEGs were screened between DB pig and Landrace pig samples, and of these, 184 DEGs were significantly up-regulated and 226 DEGs were significantly down-regulated. Through analysis of genomic data and functional analysis of DEGs, some of the important functional genes, such as CCL2, FOS, IL6, JUNB, EGR1, and GATA-6, were identified to play a potential role in the phenotypic differences between DB and Landrace pigs. The key role can lay the foundation for future breeding of new varieties that meet the needs of society.

## Abbreviations

DB: Debao black pig
DEG: differentially expressed gene
FPKM: fragments per kilobase of transcript per million mapped reads
GO: Gene Ontology
KEGG: Kyoto Encyclopedia of Genes and Genomes
LW: Large White
PPI: protein–protein interaction
qRT-PCR: quantitative real time polymerase chain reaction
SNP: single nucleotide polymorphism
